# Introducing Infectious Agents and Cancer

**DOI:** 10.1186/1750-9378-1-1

**Published:** 2006-09-14

**Authors:** Franco M Buonaguro, George K Lewis, PierGiuseppe Pelicci

**Affiliations:** 1 Viral Oncogenesis and Immunotherapy, Dpt of Experimental Oncology, Ist. Naz. Tumori "Fondazione Senatore G. Pascale, Via M. Semmola n.1, 80131 Napoli, Italy; 2 Div. of Basic Science and Vaccine Research, Institute of Human Virology, 725 W. Lombard Street, Baltimore MD 21201, USA; 3 Dpt of Experimental Oncology, European Inst. of Oncology, Via Ripamonti 435, 20141 Milan, Italy

## Abstract

*Infectious Agents and Cancer *is a new open access, peer-reviewed, online journal, which encompasses all aspects of basic, clinical and translational research that provide an insight into the association between chronic infections and cancer.

## Background

Cancer has periodically been proposed as a transmissible disease and specific pathogens have long been searched for. This proposition has been supported and confirmed in animal models, greatly contributing to our current knowledge of biology, oncology, viral oncology, genetics, and other fields. Several Nobel prizes have been awarded for research in the field of infectious diseases and cancer, including those to Johannes Andreas Grib Fibiger (1926) [1]; Peyton Rous (1966) [2]; David Baltimore, Renato Dulbecco and Howard M. Temin (1975) [3], and several other studies have been of great relevance for the field.

In 1991 Harald zur Hausen, who discovered in the early 70's the association of HPV and cervical cancer, estimated that a significant fraction (~20%) of all human cancers worldwide are associated with infections due to viruses, including human papillomaviruses (cervical cancer and other skin cancers), human T-lymphotropic viruses (adult T-cell leukemias and lymphomas in endemic areas), hepatitis B virus (liver cancer), and Epstein-Barr virus (Burkitt lymphoma and nasopharyngeal carcinoma) [4,5]. This estimate may now need to be further revised upward in light of the fact that new viral associations have been discovered and other non-viral associations have been uncovered. The list has now expanded to include a common bacterial pathogen (*Helicobacter pylori *infection with gastric carcinoma and mucosa-associated lymphoid tissue [MALT] lymphoma) [6] as well as new viruses (hepatitis C virus [HCV] with liver cancer, human herpesvirus 6 with non-Hodgkin lymphoma, human herpesvirus 8 [also known as KSHV] with Kaposi sarcoma [KS], Castleman disease, and body cavity lymphomas) [7].

Although relevant literature points out the role of pathogens in cancer and such studies have allowed fundamental discoveries in cell biology as well as in pathogenetic mechanisms, nowadays cancer etiology, in the medical community at large, mainly refers to environmental chemical contamination or some genetic predisposition, losing the connotation of a disease associated with pathogens and the possibility of a spreadable disease. Is this right?

## Infectious Diseases and Cancer

Worldwide 15 to 20% of cancers are linked to infectious diseases [4,8]. In developed Western Countries approximately 10% of all cancers are linked to infectious agents, but they account for as much as 20% of all cancers in developing countries. Such evidence contributed to the major change in cancer pathogenetic studies with no further search for specific cancer microbes, but with the identification of the oncogenic role (in a minority of infected individuals) of pathogens mainly ubiquitous in the general population. Within this new approach pathogen-related cancers have become a (final) stage in the progression of chronic infections with several scientific and practical consequences. Besides our increasing understanding of the mechanism underlying cancer development, practical outcomes of these results are improved diagnosis and identification of people at risk, the possibility to develop specific therapeutic protocols for the respective tumors, and most remarkably for cancer prevention (i.e., the preventive value of early postnatal Hepatitis B vaccination in regions with a high prevalence of Hepatitis B virus persistence).

The results achieved thus far are very promising, but a few major obstacles remain to be solved: in particular, the development of efficient therapeutic approaches for chronic infections/cancer. Multidisciplinary approaches are needed in order to complement knowledge from several disciplines such microbiology/virology, immunology, vaccinology, molecular biology, etc. in order to exploit new technologies (antigen identification, gene expression, antigen presentation, adjuvants' discovery and optimization, vaccine formulation, ex vivo DC activation, etc.).

## *Infectious Agents and Cancer *objectives

The results of relevant studies in the field should be made available to scientists and clinicians, in particular those in developing countries where the fight against pathogen-related cancers is of the utmost importance.

*Infectious Agents and Cancer *is a new open access, peer-reviewed, online journal, which encompasses all aspects of basic, clinical and translational research that provide an insight into the association between chronic infections and cancer. The data will be published with unprecedented immediacy. Every accepted manuscript will be available for reading on its date of acceptance by any person who has access to the web. Formatted versions of the articles will follow, allowing readers to view the articles in both web-friendly XML versions and typeset PDF versions – ideal for printing out.

The open access policy adopted by the journal, changes the way in which articles are published. All articles are freely and universally accessible online, and so an author's work can be read by anyone at no cost.

Moreover, the author will hold copyright for his work and grant anyone the right to reproduce and disseminate the published article, provided that it is correctly cited and no errors are introduced [9]. A copy of the full text of each article is permanently archived in an online repository separate from the journal ensuring its permanence.

*Infectious Agents and Cancer *articles are listed in PubMed and are archived in PubMed Central [10], the US National Library of Medicine's full-text repository of life science literature, and also in repositories at the University of Potsdam [11] in Germany, at INIST [12] in France and in e-Depot [13], the National Library of the Netherlands' digital archive of all electronic publications. A further benefit of open access is that the published work will likely be read by more colleagues and will be cited more highly because of the easy availability [14].

Finally, *Infectious Agents and Cancer *aims to become an exciting open forum for cancer research collecting and publishing the most innovative and significant work in the cancer field. Such results will be achieved providing not only a fast, fair, and constructive review process, with the valid support of an excellent editorial board, but also a dedicated team of scientific editors, who are willing to discuss your research, respond to your pre-submission inquiries, and ensure that all manuscripts are reviewed on the basis of scientific merit and held to the highest standards of excellence and editorial consistency.

## Closing Remarks

We would like to take this opportunity to acknowledge the *Infectious Agents and Cancer *Editorial Board  for their commitment to the journal and to express our most sincere gratitude to the entire staff of BioMed Central  for their valuable advice and support in launching this journal.

We invite you to work together with us to develop this new forum for infectious agents and cancer research, and look forward to receiving your contributions, which will help to make *Infectious Agents and Cancer *a scientific success.
